# Latin American women in dementia research: outstanding contributions, barriers, and opportunities from Argentinian, Chilean, and Colombian colleagues

**DOI:** 10.3389/fnagi.2023.1168414

**Published:** 2023-06-08

**Authors:** Sol Fittipaldi, Sandra Baez, Carolina Gonzalez-Silva, Claudia Duran-Aniotz

**Affiliations:** ^1^Latin American Brain Health Institute (BrainLat), Universidad Adolfo Ibañez, Santiago, Chile; ^2^Global Brain Health Institute (GBHI), Trinity College Dublin (TCD), Dublin, Ireland; ^3^Cognitive Neuroscience Center (CNC), Universidad de San Andres (UdeSA), Buenos Aires, Argentina; ^4^Departamento de Psicología, Universidad de los Andes, Bogotá, Colombia; ^5^Center for Social and Cognitive Neuroscience (CSCN), School of Psychology, Universidad Adolfo Ibañez, Santiago, Chile

**Keywords:** women, gender, inequity, diversity, dementia, Alzheimer’s disease, Latin America

## Abstract

Women’s contributions to science have been consistently underrepresented throughout history. Despite many efforts and some progresses being made to reduce gender inequity in science, pursuing an academic career across disciplines, including Alzheimer’s disease (AD) and other dementias, remains challenging for women. Idiosyncratic difficulties of Latin American countries likely accentuate the gender gap. In this Perspective, we celebrate outstanding contributions from Argentinian, Chilean, and Colombian colleagues in dementia research and discuss barriers and opportunities identified by them. We aim to acknowledge Latin American women’s work and bring visibility to the challenges they face throughout their careers in order to inform potential solutions. Also, we highlight the need to perform a systematic assessment of the gender gap in the Latin American dementia community of researchers.

## Introduction

Throughout the history of science, women’s contributions have been consistently underrepresented, creating a misleading and unfair mindset: women are just not interested in scientific research (Saini, 2017). Such a simplistic point of view overlooked the many cultural, religious, and social hurdles that women had to overcome to produce new knowledge, including the lack of access to higher education and gender stereotypes that cause them to be perceived as less talented for science (Leslie et al., 2015) and more inclined toward care-taking roles (Ellemers, 2018). Despite many efforts and some progresses being made to reduce gender inequity in science, pursuing an academic career remains challenging for women.

Across disciplines, the women-to-men ratio is known to decrease from early to advanced career stages (Llorens et al., 2021). Several reasons have been proposed to explain such a trend. Women are less likely to occupy key (first and last) authorship positions in high profile journals (West et al., 2013) and are cited less than men (Dworkin et al., 2020). These biases can lead to lower productivity metrics and hinder opportunities for funding acquisition and promotion to higher-level roles. Even in the case of equal outcomes, women often receive poorer evaluations of their performance (Moss-Racusin et al., 2012), fewer grants (Burns et al., 2019), and fewer prestigious awards (Lincoln et al., 2012) compared to men, indicating a systemic undervaluing of their contributions. Importantly, childcare and household responsibilities are mostly taken on by women, limiting opportunities for mobility, work presentations at scientific conferences, and networking. Taken together, these disadvantages hamper academic growth, recognition, and impact of the female scientific workforce.

This landscape is mirrored in Alzheimer’s disease (AD) and other dementias’ research. Women comprise 42.1% of all authorships in this field and only 32.8% of the last authorships (Menzel et al., 2019). Also, women have lower citation rates compared to men, publish fewer articles, and less often have prestigious authorships in collaborative papers with multiple authors (Menzel et al., 2019). Data from Alzheimer’s Research UK revealed that women hold only 33% of major grants, contrasting with 64% of successful early career grants (Andreou et al., 2022), suggesting a loss of female researchers in the junior-to-senior transition as reported for other disciplines.

The outlook for Latin American countries is even more intricate. These countries face idiosyncratic difficulties related to the scarce availability of government funds for scientific research, the political instability, and patriarchal sociocultural norms that likely accentuate the gender gap (García-Holgado et al., 2019; Silva et al., 2022). While specific data on female researchers in the dementia field across Latin America is wanting, insights from related neuroscience field reveal that less than 20% of women attain the highest position (full professor), in comparison with 30% of men, despite female neuroscientists being overrepresented at the graduate level (Silva et al., 2022). Also, Latin American neuroscientists women report higher levels of career dissatisfaction (44%) than men (28%) (Silva et al., 2021).

Beyond the inequity concerns intrinsic to these numbers, gender diversity in teams is now recognized as a key driver of creativity, innovation, and scientific discovery (Nielsen et al., 2018). Female perspectives become particularly relevant in dementia research partially because dementia affects more women than men both in terms of higher prevalence (GBD 2019 Dementia Forecasting Collaborators, 2022) and care burden (Erol et al., 2016) worldwide. Latin America is not the exception; dementia is more prevalent in women (8.97%) than men (7.26%) (Ribeiro et al., 2022), and patterns of female-to-male ratio are projected to continue (GBD 2019 Dementia Forecasting Collaborators, 2022). In Latin American countries caregiving is also disproportionally delivered by women [with numbers as high as 80% in Chile (Slachevsky et al., 2013)] who mostly experience the emotional, social, and financial costs in contexts of poor governmental support (Ibanez et al., 2021). Achieving gender equity in dementia research, specifically in highly impacted regions such as Latin America where prevalence is expected to increase 200% by 2050 (GBD 2019 Dementia Forecasting Collaborators, 2022), is necessary to amplify the voices of key actors in this global health issue.

**Figure 1:**
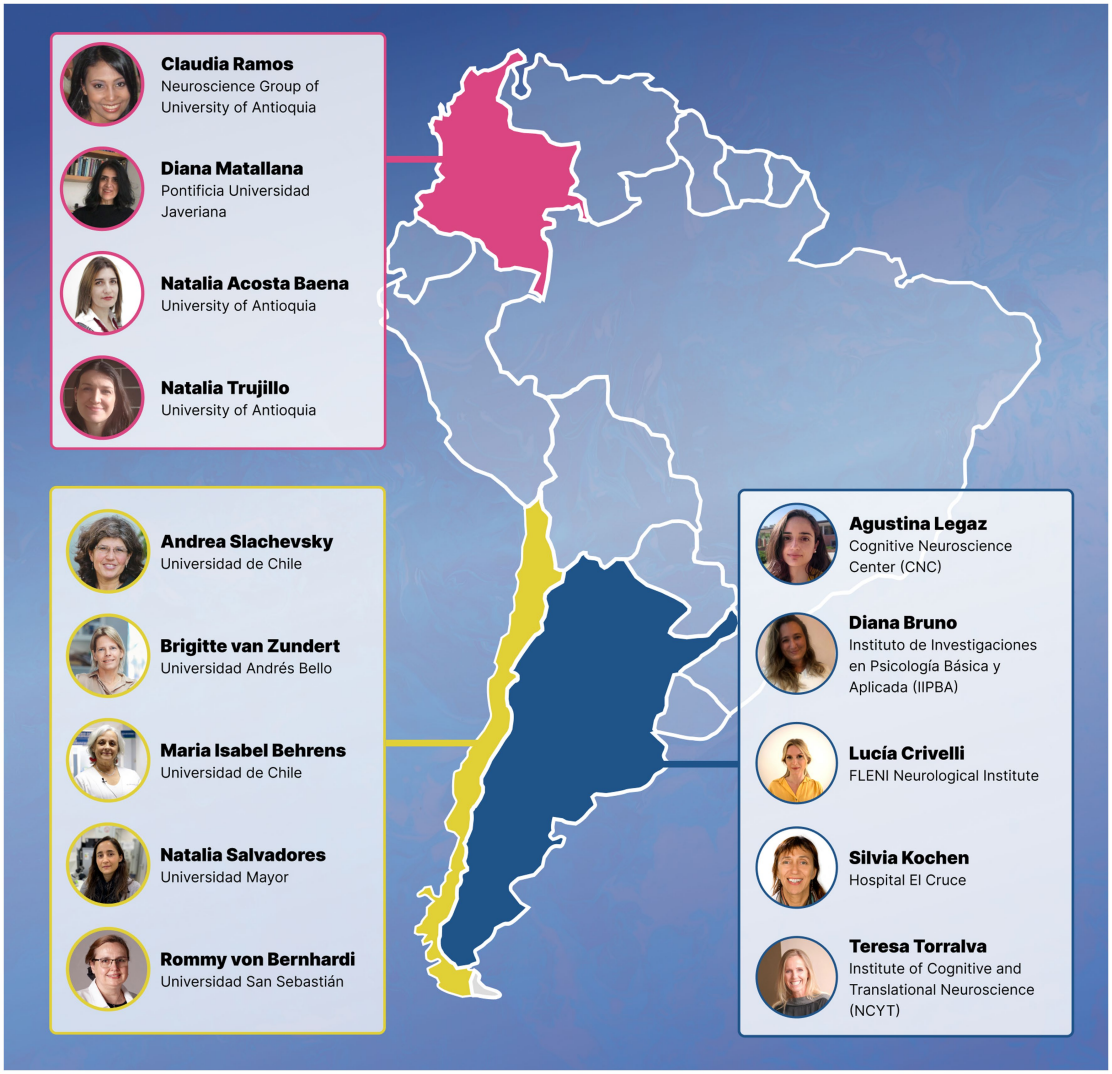
Latin American women featured in this Perspective. Female scientists who are currently developing high-impact research on Alzheimer’s Disease and other dementias in Argentina (blue), Chile (yellow), and Colombia (pink).

One of the first steps toward achieving this goal is to acknowledge women’s work and bring visibility to the challenges they face throughout their careers in order to inform potential solutions. In this Perspective, we aim to celebrate outstanding contributions from Latin American women in dementia research and discuss barriers and opportunities identified by them. We surveyed 14 national Argentinian, Chilean, and Colombian female researchers from our local networks who (a) are currently working in the region, and (b) have significantly advanced our understanding of AD and other dementias with a local impact to mitigate the burden this condition (Figure 1). We included early-, mid-career, and senior researchers focused on both basic and translational science. Their trajectories are described below, followed by a discussion of barriers, opportunities, and future directions.

### Argentina

#### Agustina Legaz, BS

Ph.D. student, Cognitive Neuroscience Center (CNC), Universidad de San Andres, Buenos Aires.

The research program of Ms. Legaz involves the study of socio-contextual modulations on memory and emotions in Latin American samples of AD, behavioral-variant frontotemporal dementia (bvFTD), and Parkinson’s disease. Her work combines novel experimental paradigms with electrophysiological and structural and functional MRI techniques. In a recent work (Legaz et al., 2022), which has been highlighted among new developments in frontotemporal dementia by the editor-in-chief of Brain journal (Husain, 2022), Legaz and colleagues showed differential multimodal deficits in the ability to learn from social feedback in AD, bvFTD, and Parkinson’s disease. In another work (Salamone et al., 2021), they showed how interoception (the sensing of visceral signals) primes the processing of emotions, and how this process is compromised in different neurodegenerative diseases. Her contributions allow a better characterization of dementia subtypes in underrepresented Latin American populations, with relevant theoretical and clinical implications in the realms of diagnosis and treatment.

#### Diana Bruno, PhD

Director, Instituto de Investigaciones en Psicología Básica y Aplicada (IIPBA), Facultad de Filosofía y Humanidades, Universidad Católica de Cuyo, San Juan.

Dr. Bruno is a neuropsychologist and researcher interested in the detection of and intervention in cognitive impairment and dementia. She has validated the Addenbrooke’s Cognitive Examination III (ACE-III), a widely used cognitive screening, for the Argentinian and Chilean populations (Bruno et al., 2020), bringing local professional communities a sensitive instrument for the detection of dementia. A review on the psychometric properties and usability of the ACE-III was critical to identify difficulties associated with its use in the region (Bruno and Schurmann Vignaga, 2019). Dr. Bruno has also validated the Cognitive Complaints Questionnaire (CCQ) aimed to assess multidomain cognitive complaints (Nuñez and Bruno, 2021). Crucially, Dr. Bruno has published “Saber Acompañar,” a toolkit to support caregivers and families of persons with dementia and other brain diseases (Torralva et al., 2021). The work of Dr. Bruno is relevant for professionals and the community. This is the result of her conviction that science must transcend academic boundaries to solve societal problems, improve quality of life, and guarantee an equative access to new developments.

#### Lucía Crivelli, PhD

Head of Neuropsychology, Department of Cognitive Neurology, Neuropsychology, and Neuropsychiatry, FLENI Neurological Institute, Buenos Aires.

Dr. Crivelli is committed with the prevention and early detection of cognitive decline and dementia in developing countries. She serves as Principal Investigator on LatAm-FINGERS, the first multi-domain lifestyle intervention in the region (Crivelli, 2020). Her role has been crucial for designing the project and reuniting the most prestigious centers and leaders from Latin America. In addition, during the last 2 years, she has studied the consequences of COVID-19 on cognitive functions in adults with no prior history of cognitive impairment, revealing that even asymptomatic cases show cognitive sequelae (Crivelli et al., 2022a). The pandemic has also driven her work on the field of tele-neuropsychology, where she published the first recommendations for Latin America, identifying barriers and challenges unique to the region and potential solutions to overcome them (Crivelli et al., 2022b).

#### Silvia Kochen, PhD

Researcher, Hospital El Cruce and National Scientific and Technical Research Council (CONICET), Buenos Aires.

Dr. Kochen’s work is focused on the study of cognition in older adults and the development of effective interventions for dementia in vulnerable populations from suburbs of Buenos Aires province. She has run a prospective longitudinal study in the memory clinic of the Neurosciences and Complex Systems Unit (EnyS, Hospital El Cruce, Florencio Varela, Buenos Aires) which revealed critical needs of users in terms of cognitive assessment and treatment (Kochen et al., 2018). Her research has also contributed to a better understanding of memory-enhancing strategies in healthy older adults (Tassone et al., 2020). Overall, Dr. Kochen’s work has implications for the development of public policies around dementia prevention, diagnosis, treatment, care, and support in populations in need from Argentina. Dr. Kochen describes herself as a “feminist scientist” committed to the integration of a gender perspective in her activities.

#### Teresa Torralva, PhD

Neuropsychologist and researcher, Institute of Cognitive and Translational Neuroscience (INCYT) and INECO Foundation, Buenos Aires.

Dr. Torralva has devoted her career to clinical neuropsychology and research, with a focus on the study of the frontal lobes and executive functions in frontotemporal dementia. In one of her most impactful publications (Torralva et al., 2009a), she showed that an Executive and Social Cognition Battery was more sensitive than classical cognitive measures to detect specific deficits in early bvFTD. This work was pioneering in revealing the relevance of ecological tests to assess early bvFTD, which can sometimes be very challenging. Dr. Torralva and her team have also developed the INECO Frontal Screening (IFS), a brief, sensitive, and specific tool to detect frontal executive impairment in neurodegeneration (Torralva et al., 2009b), which has been translated and adapted for multiple countries. Dr. Torralva has also made significant contributions in the field of social cognition in dementia, particularly showing patterns of alterations of theory of mind in bvFTD (Torralva et al., 2015). Dr. Torralva has published more than 80 papers in peer-reviewed journals, including Brain and Neuropsychologia, book chapters, and books on these topics, including “Saber Acompañar” (Torralva et al., 2021), and “Upgrade Cerebral” (Torralva, 2022).

### Chile

#### Andrea Slachevsky, PhD

Associate Professor, Faculty of Medicine, Universidad de Chile, Santiago.

Dr. Slachevsky is a neurologist specialized in cognition and dementia. She is the clinical director of the Geroscience Center for Brain Health and Metabolism (GERO) which runs the largest longitudinal cohort of aging in Latin America (Slachevsky et al., 2020). Together with health professionals and caregivers, she founded the Professional Cooperation for Alzheimer’s and Other Dementias (COPRAD), a non-profit, non-governmental organization dedicated to promoting public policies to address the needs of people living with dementia and face the challenges associated with the rapid increase of dementia prevalence. She has also participated in the preparation of the National Dementia Plan in Chile and in the creation of one of the three memory units that were implemented in the context of said Plan. Her research line focuses on neuropsychology and functionality in dementia and healthy aging. Dr. Slachevsky has developed and validated a new version of the Activities of Daily Living Questionnaire (ADLQ) that incorporates a subscale to assess the use of information and communication technologies, contributing to the evaluation of functionality in cognitive disorders (Muñoz-Neira et al., 2012). She is now working on the development of new methods to assess executive functions using serious games. Dr. Slachevsky has also participated in studies aimed to estimate the cost associated with dementia (Hojman et al., 2017) and the burden of care (Slachevsky et al., 2013) in Chile. Taken together, Dr. Slachevsky’s work is relevant for the evaluation of dementia in Latin America and the estimation of its economic impact, and has policy implications.

#### Brigitte van Zundert, PhD

Full Professor, Universidad Andrés Bello, Santiago.

Dr. van Zundert is specialized in the molecular and cellular basis of learning and memory in health and neurodegenerative diseases. The laboratory of Dr. van Zundert analyzed the PSD95 epigenetic landscape in developing hippocampus and designed a PSD95 epigenetic-targeting strategy for AD. They established that epigenetic editing of the synaptic protein PSD95 prevented and even recovered hippocampal dysfunction and memory deficits in mouse models of AD, thus validating PSD95 as a key player in memory (Bustos et al., 2017). Dr. van Zundert and colleagues have also studied the effect of urban air pollution in epigenetic gene regulation and AD hallmarks in the brain of humans and mice. They found that young healthy humans chronically exposed to urban air pollution exhibit reduced repressive epigenetic marks, increased DNA damage, and presence of AD hallmarks. In addition, mice exposed for 7 months to intermediate/high air pollution (Santiago, Chile) displayed similar brain impacts (Calderon-Garciduenas et al., 2020). These findings established the role of epigenetic mechanisms in learning and memory in neurodegenerative diseases. Dr. van Zundert has published >50 scientific papers in prestigious journals such as Brain and Neuron.

#### Maria Isabel Behrens, PhD

Full Professor, Faculty of Medicine, Universidad de Chile, Santiago.

Dr. Behrens studies the molecular mechanisms of dementia, with a focus on the inverse relationship between AD and cancer. Remarkable, using a cohort of 600 patients from the Memory and Aging Program, Dr. Behrens and his co-worker, Dr. Catherine Roe, found that AD has a protective role against the future development of cancer. Moreover, patients with cancer are apparently protected from the development of AD. This finding was replicated in the Cardiovascular Health Study–Cognition Substudy cohort with 3,000 patients and in many other publications (Roe et al., 2005). Additionally, Dr. Behrens studied the genetic cause of Kufor-Rakeb syndrome (KRS), a rare juvenile hereditary disease. KRS patients develop the typical signs of Parkinson’s disease, but also show symptoms of dementia. Dr. Behrens associated the origin of this syndrome with the loss-of-function mutation of the neuronal ATPase gene ATP13A2 (Ramirez et al., 2006). Overall, Dr. Behrens has contributed to understanding the mechanism of neurodegenerative diseases, paving the way to the development of effective therapies.

#### Natalia Salvadores, PhD

Assistant Professor, Universidad Mayor, Temuco.

The main interest of Dr. Salvadores is contributing to the understanding of neurological diseases. Her studies are focused on the molecular and cellular mechanisms underlying AD pathogenesis. Dr. Salvadores’ work involves adapting and validating protein misfolding cyclic amplification (PMCA) technique as a new potential diagnosis tool for neurodegenerative diseases. In a recent study, Dr. Salvadores was able to distinguish AD patients from control individuals with high sensitivity and specificity using cerebrospinal fluid samples (Salvadores et al., 2014). Dr. Salvadores also studied the participation of necroptosis (a programmed form of cell death) in the pathomechanism of AD. She found that oligomeric Aβ aggregates (Aβo) correlate with necroptosis activation in human AD brains. She also showed that genetic and pharmacological inhibition of necroptosis ameliorates neurodegeneration and memory loss in an AD model based on intracerebral administration of Aβo (Salvadores et al., 2022). Dr. Salvadores demonstrated for the first time the involvement of Aβ pathology in the activation of this death pathway.

#### Rommy von Bernhardi, PhD

Full Professor, Faculty of Medicine and Science, Universidad San Sebastián, Santiago.

Dr. von Bernhardi investigates the participation of glial cells in the development of neurodegenerative diseases. She is currently focused on the glial functional changes of aging that promote cytotoxic activation and neurodegeneration. She demonstrated that inflammatory stimuli induce the production of reactive oxygen species (ROS) in older mice, favoring oxidative damage during aging. The increase in ROS also induces neuroinflammation, generating a vicious circle, resulting in functional deficiencies such as memory and behavior alterations (von Bernhardi et al., 2015). She also studied the role of glial cells receptor Scavenger Receptor class A (SR-A) in AD. Dr. von Bernhardi reported the downregulation of SR-A expression in the hippocampus of aged animals and the APP/PS1 AD animal model. Additionally, Microglia and astrocytes lacking SR-A displayed impaired oxidative response and nitric oxide production (Cornejo et al., 2018). Taken together, Dr. von Bernhardi contribution allows understanding the correlation between microglial response and neurodegeneration.

### Colombia

#### Claudia Ramos, M.D.

Psychiatrist, Neuroscience Group of University of Antioquia, Medellín.

Dr. Ramos serves as psychiatrist and researcher at the mental health program for patients with neurodegenerative diseases of the Neuroscience Group of University of Antioquia. She participated in a study assessing mental disorders in young adults from families with the presenilin-1 gene mutation e280a in the preclinical stage of AD (Villalba et al., 2019). Also, she conducted a review of different genetic variants that cause AD or frontotemporal dementia in Latin America (Ramos et al., 2020). In her more recent work (Ramos et al., 2022), she investigated the association between substance use and cognitive decline in carriers of the PSEN1-E280A genetic variant showing that cigarette and alcohol were associated with an improvement of some cognitive assessments, possibly by a survival bias. Taken together, Dr. Ramos’ work helps to characterize mental health disorders in patients with dementia and develop prevention strategies.

#### Diana Matallana, PhD

Full Professor, Faculty of Medicine, Pontificia Universidad Javeriana, Bogotá.

As neuropsychologist and researcher, Dr. Matallana’s work has focused on clinical studies aimed to improve the diagnosis and treatment of neurodegenerative diseases. She also uses neuropsychological measures to study the cognitive impairments associated with psychiatric disorders and mild traumatic brain injury. One of her recent studies (Cruz-Sanabria et al., 2021) compared the neuropsychological profiles, brain morphometry, and structural connectivity patterns between patients diagnosed with bvFTD and older-age bipolar disorder patients. She has also participated in genetic studies in neurodegenerative diseases identifying 21 pathogenic variants in AD-FTLD related genes in the Colombian population (Acosta-Uribe et al., 2022). Dr. Matallana is member of the Multi-partner consortium to expand dementia research in Latin America (ReDLat), a multi-site network aimed to understand the genetic and environmental factors influencing dementia in Latin America. In a recent work of this consortium, authors reported a survey to explore the ongoing work, needs, interests, potential barriers, and opportunities for future studies related to biomarkers in the region (Parra et al., 2022). Dr. Matallana has remarkably contributed to the characterization of neuropsychological profiles and clinical and genetic assessments of Colombian patients with neurodegenerative diseases.

#### Natalia Acosta Baena, BS

Ph.D. student, Neuroscience Group of University of Antioquia, Medellín.

Ms. Acosta Baena is clinical epidemiologist, and her work is focused on the genetics of neurodegenerative diseases, mainly AD. She also investigates the relationship between neurodevelopment and dementia. She has carried out one of the largest studies assessing descendants of individuals with a mutation in presenilin 1 (PSEN1) that causes familial AD, with the aim of identifying distinct stages of clinical progression to AD dementia (Acosta-Baena et al., 2011). She participated in the Alzheimer’s Prevention Initiative Colombia Trial (Rios-Romenets et al., 2018), in which authors reported a participant retention of 94%, highlighting that this adherence plan plays a crucial role in maintaining treatment compliance and may offer guideposts for other prevention trials. Overall, Ms. Acosta Baena’s work aims to improve the quality of life of patients with dementia, clarifying the diagnosis, informing about the characteristics of these diseases, and discovering options for possible future therapies.

#### Natalia Trujillo, PhD

Full professor, University of Antioquia, Medellín.

Dr. Trujillo studies cognitive and social processing and its intervention in neurological patients and populations characterized by disruptive behavior. In ReDLat multisite project, she evaluates the role of social determinants of health on the progression of AD and is involved in the transcultural validation of functional scales. She employs neuropsychological and electrophysiological methods for assessing patients with neurodegenerative diseases. She contributed to relevant works showing early action-verb production and action semantics in patients with Parkinson’s disease (Bocanegra et al., 2015), and proposing an automated analysis of spontaneous discourse for the classification of Parkinson’s disease patients (Garcia et al., 2016). Also, she participated in a study supporting the validity of the visual short-term memory binding test and their neural correlates in the early detection of AD (Pietto et al., 2016). Her work has implications for early detection and monitoring of neurodegenerative conditions.

### Empowering Latin American women in dementia research: barriers and opportunities

In this Perspective, we celebrated the work of Latin American women in the field of dementia research. Using animal models, neuropsychological tools, and neuroimaging techniques, among others, they have made remarkable contributions to improve the characterization, assessment, diagnosis, and treatment of neurodegenerative conditions, including AD, bvFTD, and Parkinson’s disease. They have contributed to a better understanding of memory and other cognitive and sociocognitive domains in healthy adults and persons with dementia. In addition, they have characterized different genetic variants associated with dementia in Latin America, created and validated neuropsychological tests with local utility, led prevention initiatives, and developed recommendations for assessment and intervention in dementia targeting professionals, caregivers, and policy makers. Their work proves critical for the development of strategies to reduce the burden of dementia with local impact in a global context.

The barriers faced by women included in this article along their career are not essentially different from those reported in the literature. Struggles with work-life balance, childcare duties, expectations of traditional female roles, prejudice, discrimination, and difficulty in securing funding were recognized as disproportionally affecting them compared to men, as previously acknowledged by Latin American neuroscientists (Silva et al., 2021, 2022). Taken together, these barriers represent a “glass ceiling” that unequally makes harder for women to advance their careers (Cotter et al., 2001).

While much has been written about barriers of women in science, less is known about opportunities from the perspective of the persons involved. When asked to identify opportunities during their careers that empowered and energized them to stay on track, women surveyed for this article mainly mentioned: (1) working with female scientists that inspired them, (2) belonging to supportive networks that allowed them to overcome economic resources’ limitations, (3) securing funding/awards specifically tailored to women from middle-and low-income countries, and (4) being aware of increased visibility of the gender gap and actions toward reducing it at the national and international sphere. Thus, despite barriers, positive female role models, networking, and the development of policies and programs aimed at increasing women in science, in particular from underrepresented backgrounds, are identified as opportunities to change.

Regardless of the discipline, promoting the engagement and retention of women in science requires systemic changes at different levels, including individual (e.g., education, bias awareness), institutional (e.g., fair promotion and family leave policies), and cultural/societal (e.g., reduction of explicit and implicit prejudice, legislations) dimensions. While a comprehensive proposal of solutions is beyond the scope of this work and can be found elsewhere (Schrouff et al., 2019; Llorens et al., 2021; Sibener et al., 2022; Silva et al., 2022). Table 1 summarizes some potential solutions and resources to address the main barriers and opportunities that were identified by women surveyed here.

**Table 1:** Potential solutions and resources to address gender-related barriers and opportunities identified in the present work.

Barriers	Potential solutions
Struggles with work-life balance and childcare duties	Institutions should consider maintaining a flexible and family-friendly work schedule and subsume childcare expenditures during work-related travel. Institutions and conference organizers could provide free or affordable childcare options/lactation rooms. Funding agencies and reviewers should take into account parenthood and care-related delays in the academic career when assessing candidates for a job, a grant, or an award (e.g., “stop the clock” policies).
Expectations of traditional female roles, prejudice, and discrimination	All individuals should educate themselves regarding biases, stereotypes and prejudices, evaluate their own behavior, and be an active bystander against discrimination and harassment. Institutions should consider offering regular (un)conscious bias training workshops for employers and employees. Conference organizers should guarantee a balanced speaker’s gender ratio. Institutions and conference organizers should adopt zero-tolerance policies for discrimination, harassment, and abuse, and a code of conduct with clear consequences for such behaviors.
Difficulty in securing funding	Authors and scientific journals should balance gender in references’ lists to allow for more equitative academic success metrics. Funding agencies and institutions should guarantee gender balance among reviewers, editorial panels, and awardees as well as double-blind review processes. Institutions could actively outreach, suggest, and support applications for grants or promotions from female scientists.
Opportunities	Resources
Positive female role models and networking	Repositories such as Women in science, Anne’s list, and 500 Women Scientists feature female neuroscientists, allowing them to gain visibility. Networking initiatives such as the Alliance of Women Alzheimer’s Researchers (AWARE) hosted by the Alzheimer’s Association connect female researchers.
Funding/awards specifically tailored to women from middle-and low-income countries	The International Brain Research Organization (IBRO), the Society for Neuroscience (SfN), and the ALBA network offer tailored funding programs.
Visibility of the gender gap	Initiatives such as BiasWatchNeuro (a website that tracks gender diversity in neuroscience conferences), public reports of grant success rates by gender, and the use of “Inclusion and diversity statement” in manuscripts allow to oversight gender (im)balance in science.

We specifically highlight the need to perform a systematic assessment of the gender gap in the Latin American dementia community of researchers. Recent reports from the Interamerican Development Bank (López-Bassols et al., 2018) and the Latin America Regional Committee of International Brain Research Organization (IBRO LARC) and the Economic Commission for Latin America and the Caribbean (ECLAC) (Tomassini et al., 2020) offer an analysis of the gender imbalance in (neuro)science, technology, and innovation across the region. However, women’s situation specifically in the field of dementia research remains unexplored. Thus, additional work should be performed to systematically explore the participation, barriers, and opportunities of Latin American women researchers on dementia. Quantitative gender measures are needed to develop interventions aimed at reducing the gap and monitoring their impact.

The list of women featured in the present Perspective by no means intends to be exhaustive and we acknowledge that it is not representative of the entire Latin American research landscape on dementia. It consists of a selective sample from the authors’ networks in Argentina, Chile, and Colombia as a recognition of all colleagues from Latin America who are devoting their careers to advance dementia research in the region. We also acknowledge that additional disparities might contribute to women’s challenges in science, such as sexual orientation, ethnicity, religion, and socioeconomic status, among others, which need to be further examined in the Latin American context. We hope this work will inspire others and serve as a starting point to address these critical issues. Gender diversity and equity in research are critical to the global and local effort to tackle dementia.

## Data availability statement

The original contributions presented in the study are included in the article/supplementary material, further inquiries can be directed to the corresponding author.

## Ethics statement

Written informed consent was obtained from the individual(s) for the publication of any potentially identifiable images or data included in this article.

## Author contributions

SF, SB, and CD-A conceived this work and designed and performed the survey. SF wrote the manuscript in collaboration with SB, CGS, and CD-A. All authors discussed the content and substantively contributed to the revision of the manuscript.

## Funding

This work was supported by the Latin American Brain Health Institute (BrainLat). SF is an Atlantic Fellow for Equity in Brain Health at the Global Brain Health Institute (GBHI) and is supported with funding from GBHI. CD-A is supported by ANID/FONDECYT Regular 1210622, ANID/PIA/ANILLOS ACT210096, and the MULTI-PARTNER CONSORTIUM TO EXPAND DEMENTIA RESEARCH IN LATIN AMERICA [ReDLat, supported by National Institutes of Health, National Institutes of Aging (R01 AG057234), Alzheimer’s Association (SG-20-725707), Rainwater Charitable foundation, Tau Consortium, and the GBHI].

## Conflict of interest

The authors declare that the research was conducted in the absence of any commercial or financial relationships that could be construed as a potential conflict of interest.

## Publisher’s note

All claims expressed in this article are solely those of the authors and do not necessarily represent those of their affiliated organizations, or those of the publisher, the editors and the reviewers. Any product that may be evaluated in this article, or claim that may be made by its manufacturer, is not guaranteed or endorsed by the publisher.
